# Associations of the COVID-19 pandemic with quality of life: A cross-sectional study of older-age people with and without HIV in rural Uganda

**DOI:** 10.7189/jogh.13.06003

**Published:** 2023-01-20

**Authors:** Brianne Olivieri-Mui, Susanne S Hoeppner, Yao Tong, Emma Kohrt, Lien T Quach, Deanna Saylor, Janet Seeley, Alexander C Tsai, Zahra Reynolds, Samson Okello, Stephen Asiimwe, Atwiine Flavia, Ruth Sentongo, Edna Tindimwebwa, Ana-Claire Meyer, Noeline Nakasujja, Robert Paul, Christine Ritchie, Meredith Greene, Mark J Siedner

**Affiliations:** 1 Department of Health Sciences, Bouvé College of Health Sciences, Northeastern University, Boston, USA; 2 College of Science and Mathematics, University of Massachusetts Boston, Boston, USA; 3 Harvard Medical School, Boston, USA; 4 Department of Psychiatry, Massachusetts, General Hospital, Boston, USA; 5 Medical Practice Evaluation Center, Mongan Institute, Department of Medicine, Massachusetts General Hospital, Boston, USA; 6 Department of Neurology, Johns Hopkins University School of Medicine, Baltimore, USA; 7 Department of Global Health and Development, Faculty of Public Health and Policy, London School of Hygiene & Tropical Medicine, London, United Kingdom; 8 Department of Epidemiology, Harvard T.H. Chan School of Public Health, Boston, USA; 9 Gillings School of Global Public Health, University of North Carolina, Chapel Hill, USA; 10 Mbarara University of Science and Technology; 11 Kabwohe Clinical Research Centre, Kabwohe Town, Uganda; 12 Denali Therapeutics, Inc., South San Francisco, California, USA; 13 Department of Psychiatry, College of Health Sciences, Makerere University, Kampala, Uganda; 14 Department of Psychological Sciences, University of Missouri-St Louis, St. Louis, USA; 15 Division of Palliative Care and Geriatric Medicine, Department of Medicine, Massachusetts General Hospital, Boston, USA; 16 Center for Aging and Serious Illness, Mongan Institute, Department of Medicine, Massachusetts General Hospital, Boston, USA; 17 Division of Geriatrics, Department of Medicine, University of California at San Francisco, San Francisco, USA

## Abstract

**Background:**

COVID-19-related lockdowns and other public health measures may have differentially affected the quality of life (QOL) of older people with and without human immunodeficiency virus (HIV) in rural Uganda.

**Methods:**

The Quality of Life and Aging with HIV in Rural Uganda study enrolled people with and without HIV aged over 49 from October 2020 to October 2021. We collected data on COVID-19-related stressors (behavior changes, concerns, interruptions in health care, income, and food) and the participants’ QOL. We used linear regression to estimate the associations between COVID-19-related stressors and QOL, adjusting for demographic characteristics, mental and physical health, and time before vs after the lockdown during the second COVID-19 wave in Uganda. Interaction between HIV and COVID-19-related stressors evaluated effect modification.

**Results:**

We analyzed complete data from 562 participants. Mean age was 58 (standard deviation (SD) = 7); 265 (47%) participants were female, 386 (69%) were married, 279 (50%) had HIV, and 400 (71%) were farmers. Those making ≥5 COVID-19-related behavior changes compared to those making ≤2 had worse general QOL (estimated linear regression coefficient (b) = - 4.77; 95% confidence interval (CI) = -6.61, -2.94) and health-related QOL (b = -4.60; 95% CI = -8.69, -0.51). Having access to sufficient food after the start of the COVID-19 pandemic (b = 3.10, 95% CI = 1.54, 4.66) and being interviewed after the start of the second lockdown (b = 2.79, 95% CI = 1.30, 4.28) were associated with better general QOL. Having HIV was associated with better health-related QOL (b = 5.67, 95% CI = 2.91,8.42). HIV was not associated with, nor did it modify the association of COVID-19-related stressors with general QOL.

**Conclusions:**

In the context of the COVID-19 pandemic in an HIV-endemic, low-resource setting, there was reduced QOL among older Ugandans making multiple COVID-19 related behavioral changes. Nonetheless, good QOL during the second COVID-19 wave may suggest resilience among older Ugandans.

The COVID-19 pandemic interrupted community support and health care systems through lockdowns, isolation mandates, and restrictions on health care access [1]. These measures had adverse impacts in resource-constrained settings, including Uganda, where inter-generational support and access to health care differ by human immunodeficiency virus (HIV) status and across communities [1,2]. Approximately 6% of older Ugandans are living with HIV [3] and rely on access to antiretroviral therapy (ART) to maintain good health.

Interruptions in community and health care support associated with the pandemic are believed to be particularly problematic for older people, as older age groups were at highest risk for severe COVID-19 and related mortality globally [4]. People aged 45 and older make up approximately 7% of the Ugandan population and their proportion in the population is growing quickly [5], meaning more people are at risk for living with age-related declines in physical and mental health [6]. Moreover, HIV left many older people in Uganda living alone without adult children. COVID-19-related public health measures may, therefore, exacerbate issues already related to poor health outcomes (e.g. loneliness) [2]. Additionally, more than 80% of older Ugandans are informally employed in retail, trade, or manual labor, but COVID-19-restrictions limited employment options. The resulting increases in poverty have also been linked to worse health outcomes [6,7].

However, the extent to which COVID-19-related public health measures and the related behavioral responses have affected the well-being of older age adults in sub-Saharan Africa is not well known. One study among middle-aged people from Africa and the Middle East, using a psychological well-being metric for general quality of life, found significant negative associations with stress related to work, finances, home life, and COVID-19-related stress, such as being horrified, apprehensive, or feeling helpless [8]. In contrast, many African countries have faced pandemics such as Ebola and HIV, making them potentially more prepared to manage population needs during such an event [9]. Consequently, an infrastructure was developed to support HIV care facilitates engagement with the health care system, which may have mitigated the impacts of COVID-19 restrictions for older Ugandans living with HIV.

We aimed to examine general and health-related quality of life (QOL) for older Ugandans with and without HIV during the COVID-19 pandemic. We hypothesized that the response to COVID-19 related public health measures would be negatively associated with general and health-related QOL that differed based on surveys completed before or after the second set of COVID-19-related public health measures began, and that the response would be modified by HIV status.

## METHODS

### Population

The longitudinal Quality of Life and Aging with HIV in Rural Uganda (NIH R01AG059504) study [10] began collecting the first of the total four years of data from 600 older Ugandans in 2020. Due to COVID-19 restrictions, the first year of data was collected via phone calls from October 2020 to October 2021. Participants were aged ≥49 years, were attending ambulatory care at the HIV clinics at Mbarara Regional Referral Hospital and Kabwohe Immune Suppression Syndrome Clinics and were taking ART for at least three years (n = 298). For each person living with HIV enrolled, we selected a person without HIV matched by age (within quartiles), gender, and site (Mbarara or Kabwohe) from clinic catchment areas using population census data from partner studies and/or village health team lists (n = 302) [11]. The first year of data was used for this study. All study procedures were reviewed and approved by the institutional review committees at Mbarara University of Science and Technology and Mass General Brigham. We established verbal informed consent, as written consent was waived due to the COVID-19 pandemic precluding in-person data collection.

### Independent variables

Our primary independent variables of interest included a) five stressors and behaviors assessed in the survey section dedicated to participant-reported effects of COVID-19-related public health measures, b) HIV status (HIV+/HIV-), and c) whether the participant was interviewed before or after June 10, 2021, the date the Ugandan government implemented public health measures in response to the second wave of COVID-19 cases, denoted as wave 1 and wave 2 hereafter. We chose June 10, 2021, because wave 1 had largely finished at the start of data collection and we were interested in understanding if and how a government-issued lockdown might impact QOL among older-aged people in Uganda. While the wave 1 lockdown included a government-mandated ban on all travel, school closures, recommendations to limit contact with others, and, ultimately, a complete country-wide lockdown, the wave 2 lockdown was less restrictive and included 42 days of curfew and movement prohibition within or between districts for all but essential workers [12,13]. The five COVID-19-related stressor and behavioral variables of interest included: 1) the number of protective behavioral changes made in response to COVID-19 public health measures, categorized as ≤2 changes, 3-4 changes, ≥5 changes (Table S1 in the **Online Supplementary Document**); 2) a single item about their level of concern about COVID-19: “In the past 2 weeks, how concerned have you been about COVID-19 or coronavirus in your neighborhood?” (with answers “not concerned”, “somewhat concerned”, and “very concerned” available); 3) a single item on any challenges accessing health care due to COVID-19: “Over the past 2 weeks have you or any member of your household wanted to access healthcare but have been unable to do so?” (with answers “yes”, “no”, “don’t know”); 4) a single item on any challenges accessing food due to COVID-19: “Over the past 2 weeks, has your family been able to get all food and other household necessities they need?” (with answers “yes”, “no”, “don’t know”); and 5) a single item eliciting any challenges sustaining income during COVID-19: “Over the past 2 weeks, have COVID-19 laws/regulations/rules affected the ability of you or your household to earn money?” (with answers “yes”, “no”, “don’t know”). For the analysis, we combined “no” and “don’t know” responses. To check the appropriateness of including the five COVID-19 behaviors or stressors as independent items, we analyzed the variance inflation factor.

### Covariates

We included covariates based on prior literature as potential correlates of our primary outcome: age, sex, marital status (married/not married), and socio-economic factors (highest level of education, water source, and job) [2]. Although physical and mental health were not the main associations of interest, they have been shown to influence QOL [8,14], so we included depression and loneliness indicators and an adapted measure of frailty phenotype. We measured depression using the Hopkins Symptom Checklist for Depression – 20 questions (HSCL-D-20) grouped by score as little to no depression (scores <1.75), and depression (≥1.75) [15,16]. We used the University of California, Los Angeles three-item loneliness scale to measure loneliness. We split the sum of three questions (range 3-9) at the third quartile (≥5) for anyone to be considered lonely, based on previous research [17]. Frailty was measured by an adaptation of the validated Fried frailty phenotype [18]. Participants responded to five questions related to weight loss of ≥10 pounds in the prior year, mobility issues, and effort and motivation needed for activities of daily life. We categorized participants who reported deficits for at least three of five questions as frail, those reporting one to two deficits as pre-frail, and anyone reporting no deficits as robust. We measured psychosocial stress using the 10-item Perceived Stress Scale (PSS) (range 0-40) where a PSS score of ≥14 indicated “moderate to high stress” [19]. Anxiety was measured using the Generalized Anxiety Disorder 7-item (GAD-7) scale [20] (range 0-21, lower scores mean less anxiety), with scores ≥10 indicating moderate to severe anxiety. We did not use psychosocial stress and anxiety to represent mental health because they were relatively rare.

### Outcomes

Our primary outcome of interest was the validated 19-item Control, Autonomy, Self-realization and Pleasure (CASP-19) scale, a measure of general QOL that captures four domains [21]. Validation studies of the CASP-19 demonstrated Cronbach’s alphas for each domain ranging from 0.6-0.7. We summed domain scores to a continuous measure of general QOL (range 0-50, where lower values indicate worse general QOL). Our secondary outcome was health-related QOL, as measured by the validated EuroQol vertical visual analogue scale (EQ-VAS), a validated self-assessment of health-related QOL (with a range of 0-100, with 0 = worst imaginable health and 100 = best imaginable health) [19,20]. In validation studies across three samples, the Kendall’s coefficient of concordance for the EQ-VAS was 0.98 [22].

### Statistical analysis

We summarized the sample characteristics based on HIV serostatus and if the participants were interviewed in wave 1 or wave 2. We fit univariable linear regression models for each COVID-19 behavior or stressor, HIV status, COVID-19 wave, depression, loneliness, and frailty, with the primary (general QOL: CASP-19) and secondary (health-related QOL: EQ-VAS) outcomes. In models adjusted for each outcome, we included all seven independent variables and adjusted for covariates.

y = b_0_ + b_1_behavior changes + b_2_COVID concern + b_3_Food + b_4_Healthcare access + b_5_Income + b_6_HIV + b_7_COVID wave + b_8_Age + b_9_sex + b_10_marital status + b_11_Education + b_12_Water source + b_13_Job + b_14_Depression + b_15_Loneliness + b_16_Frailty

To understand if HIV status modified the association of COVID-19 behaviors or stressors with quality of life, we examined each HIV-COVID-19 behavior or stressor interaction and stratified the analysis by HIV status for significant interactions (example equation below). Analyses were conducted using R 4.1.1 and an alpha of 0.05.

y = b_0_ + b_1_behavior changes + b_2_COVID concern + b_3_Food + b_4_Healthcare access + b_5_Income + b_6_HIV + b_7_COVID wave + b_8_Age + b_9_sex + b_10_marital status + b_11_Education + b_12_Water source + b_13_Job + b_14_Depression + b_15_Loneliness + b_16_Frailty × HIV

### Post hoc analysis

In post hoc analyses, we examined the individual behavioral changes to identify those most impactful of QOL. In univariate regression, each behavioral change was examined by general and health-related QOL. We examined any behavior changes significant at the 0.05 level in univariate models in polychoric correlations to determine multicollinearity and inclusion in adjusted regression models in place of the categories of total behavior changes. We excluded behavior changes adopted among 5% or less because they produced less stable estimates.

## RESULTS

### Demographics

**Figure 1:**
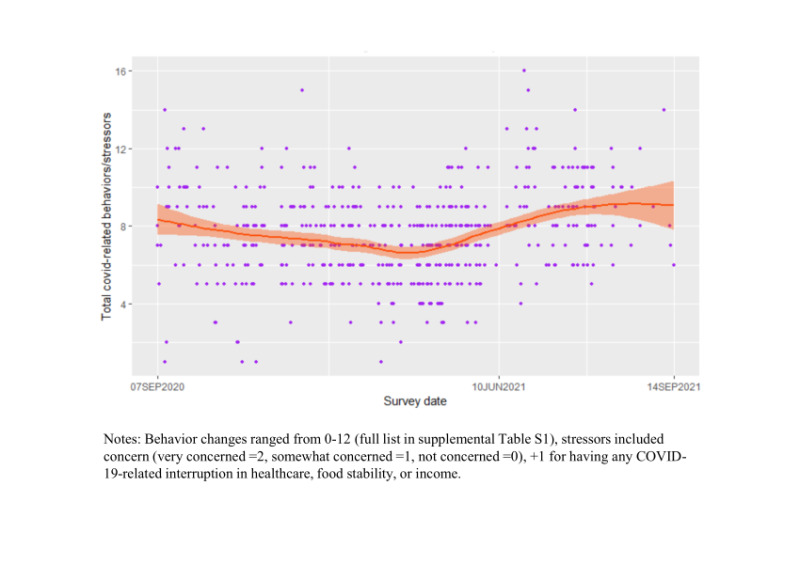
LOWESS plot of the number of COVID-related behavior changes and stressors reported by date of data collection.

We analyzed complete data from 562 participants (94% of the total cohort). Mean age was 58 years (standard deviation (SD) = 7); 265 (47%) participants were female, 386 (69%) were married, 279 (50%) had HIV, and most were farmers (n = 400 (71%)). Approximately two-thirds were surveyed before the start of the second set of COVID-19 public health measures (n = 436 (77%)). Most (n = 292 (52%)) were pre-frail, while 155 (28%) were frail. The most common comorbidities were hypertension (n = 122 (22%)), alcohol misuse (n = 56 (10%)), and high cholesterol (n = 39 (7%)). Most reported moderate psychosocial stress (n = 401 (71%)), but no anxiety (n = 404 (72%)) (**Table 1**). More than 80% were somewhat (n = 140 (25%)) or very (n = 375 (67%)) concerned about COVID-19 and everyone made at least one related behavior change; the most common were more regular hand washing (n = 533 (95%)), using a face mask (n = 520 (93%)), and avoiding crowded areas (n = 361 (64%)) and social events (n = 198 (35%)) (Table S1 in the **Online Supplementary Document**). A minority described the pandemic’s impact on health care access (n = 69 (12%)), and food (n = 97 (17%)). COVID-19 impacted income for 358 (64%) participants (**Table 2**). **Figure 1** shows the interview dates and reported cumulative COVID-19-related stressors and behavioral changes. Average general QOL was 42/50 (SD = 9); average health-related QOL was 71/100 (SD = 17) (**Table 1**).

**Table 1:** Characteristics of older Ugandans with and without HIV before and after the start of the second COVID-19 public health measures*

		HIV-		HIV+	
	**All (n = 562)**	**W1 (n = 212)†**	**W2 (n = 71)†**	**W1 (n = 224)†**	**W2 (n = 55)†**
**Age (mean (SD))**	58 (7)	59 (7)	58 (6)	58 (6)	58 (7)
**Sex**					
Female	265 (47)	102 (48)	33 (46)	104 (46)	26 (46)
**Education**					
Did not complete Primary School	288 (51)	123 (58)	31 (44)	110 (49)	24 (44)
Completed Primary School	204 (36)	67 (32)	30 (42)	83 (37)	24 (44)
Completed Secondary School or more	70 (13)	22 (10)	10 (14)	31 (14)	7 (13)
Married	386 (69)	171 (81)	56 (79)	125 (56)	34 (62)
**Job**					
Non-manual workers	89 (16)	25 (12)	7 (10)	49 (22)	8 (14)
Farm workers	400 (71)	166 (78)	52 (73)	143 (64)	39 (71)
Manual workers	67 (12)	19 (9)	11 (15)	29 (13)	8 (14)
Unemployed	6 (1)	2 (1)	1 (1)	3 (1)	0
**Water source**					
Piped into dwelling	71 (13)	25 (12)	5 (7)	39 (17)	2 (4)
Communal tap	79 (14)	37 (17)	4 (6)	34 (15)	4 (7)
Protected well	144 (26)	54 (25)	26 (37)	44 (20)	20 (36)
Protected stream	68 (12)	26 (12)	10 (14)	24 (11)	8 (15)
Public borehole	78 (14)	38 (18)	12 (17)	18 (8)	10 (18)
Other water source	122 (22)	32 (15)	14 (20)	65 (29)	11 (20)
**HIV status**					
Negative	283 (50)	212 (100)	71 (100)	0	0
Positive	279 (50)	0	0	224 (100)	55 (100)
**Frailty status**					
Robust	116 (21)	33 (16)	8 (11)	68 (30)	7 (13)
Pre-frail	291 (52)	111 (52)	53 (75)	94 (42)	33 (60)
Frail	155 (28)	68 (32)	10 (14)	62 (28)	15 (27)
**Self-reported comorbidities**					
Depression positive	199 (35)	71 (33)	37 (52)	71 (32)	20 (36)
Lonely (score ≥5)	79 (14)	25 (12)	10 (14)	37 (16)	7 (13)
Hypertension	122 (22)	65 (31)	9 (13)	42 (19)	6 (11)
Alcohol misuse positive	56 (10)	27 (13)	8 (11)	13 (6)	8 (14)
High cholesterol	39 (7)	23 (11)	4 (6)	10 (4)	2 (4)
Perceived stress (PSS)					
*Low*	154 (27)	74 (35)	8 (11)	58 (26)	0
*Moderate*	401 (71)	132 (62)	63 (89)	165 (74)	14 (25)
*High*	7 (1)	6 (3)	0	1 (0)	42 (76)
Anxiety (GAD-7)					
*None*	404 (72)	155 (73)	49 (69)	159 (71)	42 (76)
*Mild*	135 (24)	45 (21)	19 (27)	59 (26)	12 (22)
*Moderate to severe*	23 (4)	12 (6)	3 (4)	6 (3)	2 (4)
**EQ-VAS (mean (SD))**	71 (17)	67 (18)	70 (14)	75 (17)	73 (13)
**CASP-19 (mean (SD))**	42 (9)	42 (10)	43 (8)	41 (9)	43 (7)
**Pre-wave 2**	436 (77)	212 (100)	–	224 (100)	–
**Intra-wave 2**	126 (23)	–	71 (100)	–	55 (100)

HIV – human immunodeficiency virus, W1 – wave 1, W2 – wave 2, SD – standard deviation, PSS – perceived stress scale, GAD-7 – Generalized Anxiety Disorder 7-item scale, EQ-VAS – EuroQoL Visual Analog Scale measure of health-related quality of life, CASP-19 – Control Autonomy Self-realization Pleasure 19-item measure of general quality of life

*All data are presented as n (%) unless otherwise indicated. Percentages may not add to 100% due to rounding.

**†**W1 indicates the period before the start of the second COVID public health measures, while W2 indicates the period after the start of the second COVID-19 public health measures.

**Table 2:** COVID-19-related characteristics of older Ugandans with and without HIV before and after the start of the second COVID-19 public health measures

		HIV-		HIV+	
	**All (n = 562)**	W1 (n = 212)*	W2 (n = 71)*	W1 (n = 224)*	W2 (n = 55)*
**Concerned about COVID-19**					
Not much	47 (8)	26 (12)	1 (1)	20 (9)	0 (0)
Somewhat	140 (25)	53 (25)	9 (13)	72 (32)	6 (11)
Very	375 (67)	133 (63)	61 (86)	132 (59)	49 (89)
**Made any behavioral changes due to COVID-19†**					
Made <3 changes	78 (14)	43 (20)	5 (7)	25 (11)	5 (9)
Made 3-4 changes	234 (42)	103 (49)	15 (21)	92 (41)	24 (43)
Made ≥5 changes	250 (45)	66 (31)	51 (72)	107 (48)	26 (48)
**Wanted but did not get health care**					
No	493 (88)	189 (89)	52 (73)	206 (92)	46 (84)
Yes	69 (12)	23 (11)	19 (27)	18 (8)	9 (16)
**Able to get all food needed**					
No	97 (17)	32 (15)	9 (13)	43 (19)	13 (24)
Yes	465 (83)	180 (85)	62 (87)	181 (81)	42 (76)
**COVID-19 affected household money earned**					
No	204 (36)	70 (33)	6 (8)	118 (53)	10 (18)
Yes	358 (64)	142 (67)	65 (92)	106 (47)	45 (82)
**Outcome of COVID-19-related financial hardship‡**					
Not pay bills that are due	15 (3)	5 (2)	0	9 (4)	1 (2)
Take out a loan	75 (13)	25 (12)	12 (17)	28 (13)	10 (18)
Skip meals	11 (2)	2 (1)	0	9 (4)	0
Other	16 (3)	4 (2)	2 (3)	9 (4)	1 (2)
Worked more/expanded business or farming	146 (26)	64 (30)	29 (41)	34 (15)	19 (35)
Nothing	95 (17)	42 (20)	22 (31)	17 (8)	14 (25)
No financial hardship	204 (36)	70 (33)	6 (8)	118 (53)	10 (18)
**Access to soap and water at home for hand hygiene**					
No	30 (5)	6 (3)	3 (4)	14 (6)	7 (13)
Yes	531 (94)	206 (97)	68 (96)	209 (93)	48 (87)
**How often received support from friends/loved ones**					
Never	436 (78)	161 (76)	60 (85)	171 (76)	44 (80)
Once in 2 weeks	73 (13)	27 (13)	8 (11)	29 (13)	9 (16)
Once a week	37 (7)	16 (8)	3 (4)	16 (7)	2 (4)
Several times a week	14 (2)	8 (4)	0 (0)	6 (3)	0 (0)
Everyday	2 (0)	0 (0)	0 (0)	2 (1)	0 (0)
**Has ART access been impacted by COVID-19**					
Yes	–	–	–	65 (29)	40 (71)

HIV – human immunodeficiency virus, W1 – wave 1, ART – antiretroviral therapy

*W1 indicates the period before the start of the second COVID public health measures, while W2 indicates the period after the start of the second COVID-19 public health measures.

**†**A full list of behavioral changes available in Table S1 in the **Online Supplementary Document**.

‡Besides the multiple-choice answers, there were free text answers which were examined and either matched to a corresponding existing answer choice or put into “Other”; percentages may not add to 100% because of rounding.

### General QOL

In both univariable (Table S2 in the **Online Supplementary Document**) and multivariable models, general QOL was reduced for those making ≥5 COVID-19-related behavior changes relative to those making two or fewer behavioral changes (b = -4.77; 95% confidence interval (CI) = -6.61, -2.94) (**Table 3**). In post-hoc analysis, avoiding social events (b = -1.38; 95% CI = -2.65, -0.11), public transit (b = -3.16; 95% CI = -4.70, -1.62), and having younger people go to school (b = -3.78, 95%; CI = -5.48, -2.07) were the most impactful behaviors (Table S1 in the **Online Supplementary Document**). Having access to sufficient food after the start of the COVID-19 pandemic was associated with better general QOL (b = 3.10; 95% CI = 1.54, 4.66). Participants interviewed after June 10, 2021 had higher general QOL (b = 2.79; 95% CI = 1.30, 4.28), compared to participants interviewed earlier (**Table 3**). HIV did not modify the associations between COVID-19-related behaviors or stressors and QOL. Covariates were associated with worse general QOL and included older-age (b = -0.19 per year; 95% CI = -0.28, -0.10), getting water from a protected well compared to getting it piped into the dwelling (b = -2.08; 95% CI = -4.13, -0.02), depression (b = -7.02, 95% CI = -8.47, -5.58), loneliness (b = -5.69; 95% CI = - 7.49, -3.89), and being pre-frail (b = -2.30; 95% CI = -3.95, -0.64) compared to being robust.

**Table 3:** Adjusted estimates of the change in mean CASP-19 and EQ5-VAS QOL scores

	Primary		Secondary	
	**CASP-19 general QOL estimate (95% CI)**	***P*-value**	**EQ-VAS health-related QOL estimate (95% CI)**	***P*-value**
Age	-0.19 (-0.28, -0.10)	<0.01	-0.35 (-0.55, -0.15)	<0.01
**Sex**				
Female	-1.34 (-2.75, 0.08)	0.07	-1.12 (-4.28, 2.03)	0.49
Male	Ref		ref	
**Married**				
Married	1.37 (-0.15, 2.89)	0.08	-0.45 (-3.83, 2.93)	0.80
Not married	Ref		Ref	
**Education**				
Completed primary school	1.05 (-0.23, 2.32)	0.12	1.09 (-1.75, 3.92)	0.45
Completed secondary school or more	0.93 (-1.13, 3.00)	0.38	-2.90 (-7.49, 1.68)	0.21
Did not complete primary school	Ref		Ref	
**Water source**				
Communal tap	-1.04 (-3.27, 1.19)	0.36	-2.62 (-7.58, 2.33)	0.30
Protected well	-2.08 (-4.13, -0.02)	0.05	-3.54 (-8.11,1.03)	0.13
Protected stream	-1.97 (-4.33, 0.39)	0.10	-1.75 (-7.00, 3.51)	0.51
Public borehole	-1.08 (-3.35, 1.18)	0.35	-2.29 (-7.33, 2.74)	0.37
Other water source	-1.21 (-3.24, 0.81)	0.24	-2.11 (-6.62, 2.40)	0.36
Piped into dwelling	Ref		Ref	
**Job**				
Manual worker	0.98 (-0.90, 2.86)	0.31	2.98 (-1.20, 7.16)	0.16
Non-Manual worker	-0.59 (-2.35, 1.17)	0.51	-0.93 (-4.84, 2.98)	0.64
Unemployed	-2.98 (-8.56, 2.59)	0.29	-10.34 (-22.74, 2.07)	0.10
Farmer	Ref		Ref	
**HIV+**	0.04 (-1.20, 1.28)	0.95	5.67 (2.91, 8.42)	<0.01
**COVID-19 behaviors**				
Total behavior changes				
*3-4 changes*	-1.52 (-3.31, 0.26)	0.09	-3.04 (-7.01, 0.92)	0.13
*≥5 changes*	-4.77 (-6.61, -2.94)	<0.01	-4.60 (-8.69, -0.51)	0.03
*≤2 changes*	Ref		Ref	
COVID-19 concern				
*Somewhat concerned*	-0.45 (-2.76, 1.86)	0.70	1.92 (-3.22, 7.05)	0.46
*Very concerned*	0.79 (-1.38, 2.97)	0.48	1.75 (-3.08, 6.58)	0.48
*Not concerned*	Ref		Ref	
COVID-19 had access to food	3.10 (1.54, 4.66)	<0.01	1.99 (-1.47, 5.46)	0.26
COVID-19 impacted health care access	0.65 (-1.16, 2.46)	0.48	2.03 (-1.99, 6.06)	0.32
COVID-19 impacted income	1.29 (-0.04, 2.62)	0.06	-1.08 (-4.04, 1.87)	0.47
**Mental health**				
Depression	-7.02 (-8.47, -5.58)	<0.01	-11.53 (-14.75, -8.31)	<.001
Loneliness	-5.69 (-7.49, -3.89)	<0.01	-5.58 (-9.59, -1.57)	<0.01
**Frailty status**				
Pre-frail	-2.30 (-3.95, -0.64)	<0.01	-2.95 (-6.64, 0.73)	0.12
Frail	-1.43 (-3.11, 0.24)	0.09	-0.86 (-4.58, 2.86)	0.65
Robust	Ref		Ref	
COVID-19 W2*	2.79 (1.30, 4.28)	<0.01	3.42 (0.11, 6.74)	0.04

HIV – human immunodeficiency virus, CI – confidence interval, EQ-VAS – EuroQoL Visual Analog Scale measure of health-related quality of life, CASP-19 – Control Autonomy Self-realization Pleasure 19-item measure of general quality of life, QOL – quality of life, W2 – wave 2, Ref – reference

*Indicates participants interviewed after the start of the second COVID-19 public health measures.

### Health-related QOL

Health-related QOL was lower for those making ≥5 COVID-19-related behavior changes (b = -4.60; 95% CI = -8.69, -0.51) (**Table 3** and Table S2 in the **Online Supplementary Document**). In the post-hoc analysis, using hand sanitizer (b = 3.79; 95% CI = 0.79, 6.78) was the most impactful behavior (Table S1 in the **Online Supplementary Document**). Participants interviewed after June 10, 2021 had higher health-related QOL (b = 3.42; 95% CI = 0.11, 6.74). Covariates associated with worse health-related QOL included older-age (b = -0.35 per year; 95% CI = -0.55, -0.15), depression (b = -11.53; 95% CI = -14.75, -8.31), and loneliness (b = -5.58; 95% CI = -9.59, -1.57). Having HIV was associated with better health-related QOL (b = 5.67; 95% CI = 2.91, 8.42). For the secondary health-related QOL outcome, the interaction between HIV and the COVID-19 impact on health care pointed to modification; among people without HIV, having sufficient access to health care was associated with better health-related QOL (b = 5.65; 95% CI = 0.23, 11.08), but was not significant among people with HIV (**Table 4**).

**Table 4:** Adjusted estimates of the change in mean EQ-VAS score for health-related quality of life stratified by HIV status

	HIV+ estimate (95% CI)	*P*-value	HIV- estimate (95% CI)	*P*-value
**Intercept**	112.77 (92.13, 133.42)		86.35 (63.48, 109.23)	
**Age**	-0.38 (-0.66, -0.09)	0.01	-0.30 (-0.59, -0.00)	0.05
**Sex**				
Female	-0.55 (-5.24, 4.14)	0.82	-1.05 (-5.67, 3.57)	0.66
Male	Ref		Ref	
**Married**				
Married	-1.37 (-5.80, 3.05)	0.54	2.74 (-2.74, 8.21)	0.33
Not married	Ref		Ref	
**Education**				
Completed primary school	0.28 (-3.58, 4.15)	0.89	0.78 (-3.54, 5.09)	0.72
Completed secondary school or more	-6.19 (-12.46, 0.08)	0.05	0.23 (-6.79, 7.26)	0.95
Did not complete primary school(ref)	Ref		Ref	
**Water source**				
Communal tap	-1.67 (-8.47, 5.13)	0.63	-3.55 (-11.02, 3.93)	0.35
Protected well	-4.76 (-11.11, 1.59)	0.14	-1.88 (-8.61, 4.86)	0.58
Protected stream	-4.16 (-11.31, 2.99)	0.25	0.95 (-7.02, 8.93)	0.81
Public borehole	-5.36 (-12.86, 2.14)	0.16	0.20 (-6.95, 7.34)	0.96
Other water source	-4.49 (-10.34, 1.36)	0.13	2.57 (-4.85, 9.99)	0.50
Piped into dwelling	Ref		Ref	
**Job**				
Manual worker	1.47 (-3.94, 6.88)	0.59	2.99 (-3.69, 9.66)	0.38
Non-Manual worker	-3.23 (-8.31, 1.84)	0.21	2.62 (-3.74, 8.98)	0.42
Unemployed	-13.03 (-30.61, 4.54)	0.15	-9.00 (-27.48, 9.48)	0.34
Farmer	Ref		Ref	
**COVID-19 behaviors**				
Total behavior changes				
*3-4 changes*	-1.19 (-7.32, 4.94)	0.70	-4.65 (-10.14, 0.84)	0.10
*≥5 changes*	-1.87 (-8.01, 4.27)	0.55	-8.23 (-14.02, -2.43)	<0.01
*≤2 changes*	Ref		Ref	
COVID-19 concern				
*Somewhat concerned*	-4.19 (-11.81, 3.44)	0.28	5.60 (-1.87, 13.07)	0.14
*Very concerned*	-3.31 (-10.58, 3.96)	0.37	4.89 (-1.84, 11.62)	0.15
*Not concerned*	Ref		Ref	
COVID-19 had access to food	1.61 (-3.07, 6.29)	0.50	3.22 (-2.22, 8.65)	0.25
COVID-19 impacted health care access	-4.49 (-10.75, 1.78)	0.16	5.65 (0.23, 11.08)	0.04
COVID-19 impacted income	-1.03 (-5.12, 3.06)	0.62	-0.71 (-5.17, 3.74)	0.75
**Mental health**				
Depression	-13.24 (-17.94, -8.53)	<0.01	-10.27 (-14.87, -5.67)	<0.01
Loneliness	-2.91 (-8.24, 2.42)	0.28	-7.37 (-13.84, -0.90)	0.03
**Frailty status**				
Pre-frail	-3.18 (-8.12, 1.76)	0.21	-2.75 (-8.55, 3.05)	0.35
Frail	-0.86 (-5.67, 3.94)	0.72	-0.83 (-6.95, 5.30)	0.79
Robust	Ref		Ref	
COVID-19 W2*	1.47 (-3.26, 6.19)	0.54	5.48 (0.56, 10.41)	0.03

HIV – human immunodeficiency virus, CI – confidence interval, ref – reference category, W2 – wave 2, EQ-VAS – EuroQoL Visual Analog Scale measure of health-related quality of life

*Indicates participants interviewed after the start of the second COVID-19 public health measures.

## DISCUSSION

We observed a reduction in general QOL for older-aged Ugandans practising the greatest number of COVID-19 protective health behaviors during the pandemic. We also found that food security was strongly associated with better QOL in this population during the pandemic, consistent with other studies in South Africa [23]. Similarly, we found significant associations between mental health conditions and QOL. However, contrary to our hypothesis, we found that people interviewed after the onset of the public health measures related to the second COVID-19 wave had better QOL, while HIV rarely modified QOL.

Two of the five COVID-19 behaviors and stressors we considered were solitarily associated with QOL measures: making behavioral changes and having COVID-19 interrupt food security. The adoption of five or more protective behavioral changes was associated with reduced general and health-related QOL and may indicate a link between participants’ underlying coping style reflected as behavior changes; the most associated behavior changes were using hand sanitizer and avoiding social events, public transit, greeting other people, and sending young family to school. Similar findings were demonstrated in Middle East and North African populations, where from 35%-46% of participants had moderate to severe psychological effects due to COVID-19 and demonstrated avoidance behaviors [8]. A South African study found increases in similar behaviors such as avoiding transportation and crowds, and using a face mask [24]. Moreover, acquiring sufficient food means going to markets, potentially using transit, and interacting with people in similar settings to a crowd or social event. Therefore, we hypothesize that the behavior changes may have impacted the ability to acquire sufficient food during the COVID-19 pandemic and subsequently reduced QOL. In sub-Saharan Africa, food insecurity is more likely to be experienced by older people, particularly women [25,26], but is dependent on many personal and environmental factors such as household size, finances, employment status, and supply [27]. Most of our participants worked as farmers and many (26%) expanded their farming in response to hardship (**Table 2**), which is consistent with the reported 3% increase in crop farming in Uganda during the pandemic [28]. For farmers, crops could be consumed or sold and therefore may represent having sufficient access to food and averting the decrease in QOL that might otherwise be associated with the COVID-19 interruptions in income.

Older aged Ugandans had worse general and health-related QOL than the relatively younger ones in our sample. Previous research has linked stress and morbidity with older ages and being female [29,30]. Moreover, high demand on older people to be the providers for their extended family [29] and limited resources (e.g. time, money) or distance to facilities [31] can preclude health care-seeking in non-pandemic times. This may be one reason that health-related QOL seemed to improve among people without HIV who had COVID-19 interruptions in health care; they are inherently healthier and in lesser need of medical interventions. Moreover, general QOL for our sample did not seem to be associated with COVID-19-related interruptions in health care potentially reflecting a mitigating effect of the creation of innovative ART refill and delivery programs [32] or that social support, such as might come from engaging with the HIV care community, benefits QOL [33].

Historically, pandemic emergencies that involve quarantine have been associated with stress disorder, particularly among younger-age groups [34]. We found better QOL after the start of the second COVID-19 lockdown. We hypothesize that this might be due to the timing of data collection. Our study took place before and during the second wave, which was associated with a limited lockdown that was much less restrictive measures than the first one, which included orders to avoid hugging or touching people outside the household, a ban on international and domestic travel, total school closures, and ultimately a complete country wide lockdown [12,13]. Our study was delayed by the first lockdown, so we were unable to capture data during that event. Other studies in South Africa, the Middle East, and Northern Africa have demonstrated pervasive pandemic-related anxiety, fear, panic, and stress, but were conducted earlier in 2020 than we collected data [8,35]. We suspect that any major association between lockdown and QOL in our sample would have been more likely experienced during that first wave, and the milder restrictions put in place during the second wave were both less deleterious and experienced through the lens of recent history. A study of COVID-19 effects across three South African provinces confirmed this finding by showing that the proportion of people with depression and anxiety decreased or remained relatively low from April to December 2020 [24].

Finally, we found that mental health conditions, including depression and loneliness, were significantly associated with both health related and general QOL. This is consistent with research in older populations in western countries [36]. Notably, only 14% of our sample was considered lonely, but the deleterious effects of loneliness have been researched extensively [37,38]; older Ugandans do not seem to be exceptions. Depression was present in 35% of the sample, while we found people with HIV had less depression (32.5%) than people without HIV (38%). This contradicts US reports [39] and may reflect a benefit of the community created by HIV care programs in Africa. Our findings advance our understanding by suggesting the potential for specific mental health deficits making up the relationship between mental health and QOL.

### Limitations

This study has several limitations. Because our data are cross-sectional, we cannot make causal inferences about the relationship between QOL, mental health, and COVID-19-related behaviors. Moreover, the pandemic’s nature means reverse causation is possible where worse QOL could result in more COVID-19-related stressors and behavior changes. All data were collected via self-report through phone calls because of COVID-19 travel restrictions. Therefore, recall and social desirability bias may be present, though we attempted to mitigate this by training our research staff in data collection. Moreover, our sample was limited to people living with HIV in care for a minimum of three years, so our findings are not representative for people living with HIV not on ART outside the care system. However, with the widespread rollout of ART across the region, most people living with HIV are now accessing therapy [3], making our sample reflective of a treated HIV population.

## CONCLUSIONS

We found that an accumulation of COVID-19-related behavioral changes and stressors, food security, and a variety of mental health conditions were associated with QOL among older people in Uganda. We also saw improved QOL after the start of the second COVID-19 wave, potentially reflecting past experiences with more severe lockdown restrictions. Based on our findings, targets for preserving QOL in future pandemics might include focusing on behavior changes, food insecurity, and people experiencing lockdown-related loneliness or depression. Moreover, future research should explore resilience across COVID-19 waves more deliberately, as it may be instructive for future public health crises that put older people at risk.

## Additional material


Online Supplementary Document
