# Supplementary material for: Associations of the COVID-19 pandemic with quality of life: A cross-sectional study of older-age people with and without HIV in rural Uganda
Source: J Glob Health. 2023 Jan 20;13:06003. doi: 10.7189/jogh.13.06003 (PMC9850875; doi:10.7189/jogh.13.06003)
Supplement: Online Supplementary Document [file jogh-13-06003-s001.pdf]

ONLINE SUPPLEMENTARY DOCUMENT

Title: Associations of the COVID-19 pandemic on quality of life: cross-sectional study of older-age people with and without HIV in rural Uganda

Authors: Brianne Olivieri-Mui[1], Susanne S. Hoeppner[2,3], Yao Tong[2,3], Emma Kohrt[1], Lien T. Quach[2,3], Deanna Saylor[4], Janet Seeley[5], Alexander C. Tsai[2,3], Zahra Reynolds[2], Samson Okello[6,7], Stephen Asiimwe[2,6,8], Atwiine Flavia[6], Ruth Sentongo[6], Edna Tindimwebwa[8], Ana-Claire Meyer[4], Noeline Nakasujja[9], Robert Paul[10], Christine Ritchie[2,3], Meredith Greene[11], Mark J. Siedner[2,3,6]

Affiliations:[1]Northeastern University, [2]Massachusetts General Hospital and University of Massachusetts Boston, [3]Harvard Medical School, [4]Johns Hopkins University School of Medicine, [5]London School of Hygiene & Tropical Medicine, [6]Mbarara University of Science and Technology, [7]University of North Carolina at Chapel Hill, [8]Kabwohe Clinical Research Centre, [9]Makerere University, [10]University of Missouri-St Louis, [11]University of California, San Francisco

Table S1. Specific COVID-related behavioral changes made and post hoc analysis of the association of each with quality of life.

	All	CASP-19			EQ-VAS		
	(n=562) n(%)	**Univariate p-value**	**Adjusted[a] Estimate (95%CI)**	**p-value**	**Univariate p-value**	**Adjusted[a] Estimate (95%CI)**	**p-value**
Changes made include:							
No changes	0	-			-		
Wash hands more often	533 (95)	0.678			0.918		
using face mask	520 (93)	0.429			0.606		
Avoided crowded areas	361 (64)	0.501			0.950		
Avoid social events	198 (35)	**0.005**	**-1.38 (-2.65 – -0.11)**	**0.033**	0.615		
using hand sanitizer	141 (25)	**0.032**	0.74 (-0.60 – 2.07)	0.280	**0.000**	3.79 (0.79 – 6.78)	0.013
avoid taking public transit	111 (20)	**0.000**	**-3.16 (-4.70 – -1.62)**	**<0.001**	**0.004**	-2.88 (-6.15 – 0.39)	0.084
avoided hugging	99 (18)	0.056			0.865		
avoided having children/ young adults going to school	80 (14)	**0.011**	**-3.78 (-5.48 – -2.07)**	**<0.001**	0.893		
avoided greeting other people	71 (13)	0.000	-1.68 (-3.40 – 0.03)	0.054	0.856		
avoided travelling long distances	69 (12)	0.076			0.278		
avoided touching face	45 (8)	0.085			0.058		
avoided going outside	38 (7)	0.827			0.070		
avoided people who present symptoms	27 (5)	0.976			0.507		
Avoided family members/ friends	26 (5)	0.032			0.234		
avoided going to work	18 (3)	0.079			0.000		
self-isolation	15 (3)	0.024			0.506		
wearing gloves	7 (1)	0.985			0.997		
avoided seeking healthcare for preventive care	3 (1)	0.723			0.826		
avoided seeking healthcare for illness	2 (0)	0.027			0.750		
avoided picking up meds for chronic disease	0	-			-		
living elsewhere	0	-			-		
Other changes	65 (12)	0.891			0.443		

Note: [a]Model adjusted for age, sex, education, water source, job, HIV status, COVID concern, COVID food security, COVID healthcare seeking, COVID income interruption, depression, loneliness, frailty, and COVID wave.

Table S2. Univariate associations of independent variables, HIV status, and Wave with the primary and secondary QOL outcomes

	Primary	*Secondary*
	CASP-19 *Estimate (95% CI)*	*EQ5-VAS* *Estimate (95% CI)*
COVID behaviors		
Total behavior changes		
3-4 changes	-0.26 (-2.57 – 2.04)	-0.22 (-4.59 – 4.15)
5+ changes	-4.10 (-6.38 – -1.82)	-3.08 (-7.41 – 1.26)
<3 changes	ref	ref
COVID concern		
Somewhat concerned	-1.35 (-4.38 – 1.67)	2.24 (-3.41 – 7.90)
Very concerned	0.62 (-2.16 – 3.40)	2.20 (-2.98 – 7.39)
Not concerned	ref	Ref
COVID had access to food	6.32 (4.38 – 8.26)	5.41 (1.70 – 9.13)
COVID impacted healthcare access	-1.35 (-3.65 – 0.95)	-2.11 (-6.39 – 2.17)
COVID impacted income	-0.69 (-2.27 – 0.89)	-4.49 (-7.41 – -1.58)
Mental Health index	-4.77 (-5.48 – -4.07)	-6.41 (-7.82 – -5.01)
Frailty status		
Pre-frail	-6.36 (-8.26 – -4.46)	-10.31 (-13.88 – -6.75)
Frail	-2.86 (-4.98 – -0.73)	-3.81 (-7.80 – 0.18)
Robust	ref	ref
COVID wave 2	1.5 (-0.31 – 3.32)	0.62 (-2.76 – 4.00)
HIV+	-0.62 (-2.14 – 0.90)	6.36 (3.58 – 9.13)
